# Time reversal symmetry-protected transport at correlated oxide interfaces

**DOI:** 10.1093/nsr/nwaf156

**Published:** 2025-05-07

**Authors:** Mengke Ha, Qing Xiao, Zhiyuan Qin, Dawei Qiu, Longbing Shang, Xinyi Liu, Pu Yan, Changjian Ma, Danqing Liu, Chengyuan Huang, Zhenlan Chen, Haoyuan Wang, Chang-Kui Duan, Zhaoliang Liao, Wei-Tao Liu, Yang Gao, Kecheng Cao, Jiangfeng Du, Guanglei Cheng

**Affiliations:** CAS Key Laboratory of Microscale Magnetic Resonance and School of Physical Sciences, University of Science and Technology of China, Hefei 230026, China; CAS Key Laboratory of Microscale Magnetic Resonance and School of Physical Sciences, University of Science and Technology of China, Hefei 230026, China; CAS Key Laboratory of Microscale Magnetic Resonance and School of Physical Sciences, University of Science and Technology of China, Hefei 230026, China; Hefei National Laboratory, University of Science and Technology of China, Hefei 230026, China; CAS Key Laboratory of Microscale Magnetic Resonance and School of Physical Sciences, University of Science and Technology of China, Hefei 230026, China; CAS Key Laboratory of Microscale Magnetic Resonance and School of Physical Sciences, University of Science and Technology of China, Hefei 230026, China; Physics Department, State Key Laboratory of Surface Physics, Key Laboratory of Micro and Nano Photonic Structures, Fudan University, Shanghai 200433, China; School of Physical Science and Technology & Shanghai Key Laboratory of High-resolution Electron Microscopy, ShanghaiTech University, Shanghai 200093, China; CAS Key Laboratory of Microscale Magnetic Resonance and School of Physical Sciences, University of Science and Technology of China, Hefei 230026, China; CAS Key Laboratory of Microscale Magnetic Resonance and School of Physical Sciences, University of Science and Technology of China, Hefei 230026, China; CAS Key Laboratory of Microscale Magnetic Resonance and School of Physical Sciences, University of Science and Technology of China, Hefei 230026, China; CAS Key Laboratory of Microscale Magnetic Resonance and School of Physical Sciences, University of Science and Technology of China, Hefei 230026, China; Hefei National Laboratory, University of Science and Technology of China, Hefei 230026, China; CAS Key Laboratory of Microscale Magnetic Resonance and School of Physical Sciences, University of Science and Technology of China, Hefei 230026, China; CAS Key Laboratory of Microscale Magnetic Resonance and School of Physical Sciences, University of Science and Technology of China, Hefei 230026, China; Hefei National Laboratory, University of Science and Technology of China, Hefei 230026, China; National Synchrotron Radiation Laboratory, University of Science and Technology of China, Hefei 230026, China; Physics Department, State Key Laboratory of Surface Physics, Key Laboratory of Micro and Nano Photonic Structures, Fudan University, Shanghai 200433, China; Department of Physics, University of Science and Technology of China, Hefei 230026, China; School of Physical Science and Technology & Shanghai Key Laboratory of High-resolution Electron Microscopy, ShanghaiTech University, Shanghai 200093, China; CAS Key Laboratory of Microscale Magnetic Resonance and School of Physical Sciences, University of Science and Technology of China, Hefei 230026, China; Hefei National Laboratory, University of Science and Technology of China, Hefei 230026, China; Institute of Quantum Sensing and School of Physics, Zhejiang University, Hangzhou 310027, China; CAS Key Laboratory of Microscale Magnetic Resonance and School of Physical Sciences, University of Science and Technology of China, Hefei 230026, China; Hefei National Laboratory, University of Science and Technology of China, Hefei 230026, China

**Keywords:** LaAlO_3_/SrTiO_3_, correlated oxide interface, quantum oscillation, time-reversal symmetry, ferroelastic domain wall

## Abstract

Time-reversal symmetry (TRS) protection is core to topological physics, yet its role in correlated oxides—typically non-topological—remains underexplored. This limit hampers the potential in engineering exotic quantum states by fusing TRS protection and rich emergent phenomena in the oxide platform. Here, we report evidence of a TRS-protected subband at oxygen vacancy-free LaAlO_3_/SrTiO_3_ interfaces. This subband causes a low-field quantum oscillation with anomalous characteristics: exceptionally light electron mass, aperiodicity, and susceptibility to magnetic fields. All findings align with a Rashba model in which TRS-protected transport occurs along quasi-1D ferroelastic domain walls, which possess a Dirac band topology and a giant Rashba spin-orbit coupling, two orders stronger than the 2D interface. Our results deepen the understanding of SrTiO_3_-based electron systems, unveiling an appealing new platform for quantum engineering.

## Teaser

A low-field anomalous quantum oscillation is discovered at ultra-clean LaAlO3/SrTiO3 interfaces, which is attributed to the time-reversal symmetry-protected transport along quasi-1D ferroelastic domain walls.

## INTRODUCTION

Time-reversal symmetry (TRS) is fundamental to many novel physical phenomena, including topological surface states [1,2], quantum magnetism [3], and unconventional superconductivity [4]. It protects transport from local perturbations by imposing spin-momentum locking in quantum materials with strong spin-orbit coupling (SOC) [5,6]. It is, therefore, a crucial ingredient in engineering quantum states of matter, e.g. the Majorana zero mode [7]. Meanwhile, correlated oxide interfaces are widely regarded as a versatile quantum engineering platform with abundant emergent phenomena [8–11]. Despite their promise, realizing TRS-protected transport in these materials has proven elusive [12], as opposed to simple binary compounds and 2D materials [13]. Although large Rashba SOC is indeed present at oxide interfaces due to inversion symmetry breaking [14], the TRS protection is often hampered by strong electron-electron interactions and high defect concentrations in oxides. Achieving TRS protection in correlated oxides is thus highly appealing in quantum engineering; however, it continues to pose significant experimental challenges.

A viable approach to this challenge is to explore quasi-1D oxide channels with strong SOC and enhanced sample quality. The reduced dimension in 1D will more effectively suppress backscattering paths with spin-momentum locking under TRS. Here in this work, we show evidence of TRS-protected transport on quasi-1D ferroelastic domain walls (FDWs) at ultraclean LaAlO_3_/SrTiO_3_ (LAO/STO) oxide interfaces. Notably, we report the observation of an anomalous light subband at modulation-doped 2D LAO/STO interfaces. The associated effective mass is remarkably light compared to the typical 3*d* orbitals in STO-based electron systems. We investigate the induced quantum oscillations and back-gated multiband transport and reveal anomalous characteristics of this subband, including aperiodicity and susceptibility to the external magnetic field. These results are consistent with TRS-protected transport on quasi-1D FDWs with a nontrivial band topology. Finally, we explore the connection between superconductivity and quantum oscillations, suggesting the possible existence of a quantum critical point.

## INTERFACE ENGINEERING

FDWs are ubiquitously formed in STO due to the structural phase transition at *T* = 105 K. Spectroscopic and imaging studies have proven their existence and revealed the strain-tunable polarity [15,16], higher conductivity [17], and more robust superconductivity than the surrounding bulk [18]. It is intuitive to think that these 1D channels may have stronger SOC due to larger polarization on FDWs and thus be a natural place to study TRS-related physics. However, the impact of FDWs on electrical transport has rarely been observed in STO-based electron systems, possibly due to the presence of a large conducting background and oxygen vacancy (V_O_) trapping on FDWs which reduce SOC strength [19]. In addition, complex oxide interfaces are usually filled with defects compared to semiconductor heterostructures [20]. At the LAO/STO interface, oxygen vacancies (V_O_), strontium vacancies (V_Sr_), and ion intermixing constitute a comprehensive defect structure limiting mobilities beyond 10 000 cm^2^/Vs.

**Figure 1.**
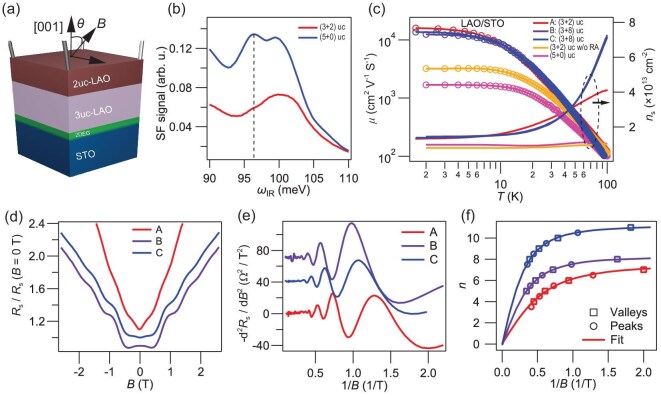
Interface design and anomalous quantum oscillations. (a) Schematic illustration of the sample layout. (b) SF spectra of a (5 + 0) sample (blue) and a (3 + 2) sample (red) suggest oxygen vacancies at the interface of (3 + 2) sample are suppressed. (c) Temperature dependence of carrier density and low-field (*B* = 0.5 T) mobility (weighted average of all subbands). The modulation-doped samples (A, B, and C) show the highest mobilities. (d) The normalized sheet resistance of three samples (A, B, and C). (e) Aperiodic quantum oscillations in the 1/*B* axis. These oscillations start at a low field ∼0.3 T with decreasing amplitudes at high fields. (f) Nonlinear Landau fan diagram. Solid lines are fittings to Eq. (1); open rectangles and circles are valleys (integer) and peaks (half integer) of ${R_{\mathrm{S}}}$, respectively. The nonlinearity indicates the 1D to 3D transition of the transport on the FDWs.

We design (*m* + *n*) uc LAO/STO interfaces to routinely achieve unprecedented mobilities over 10 000 cm^2^/Vs in pristine samples (Fig. 1(a)). Such sample configuration facilitates a modulation-doping mechanism [21,22]: the interfacial buffer LAO layer (*m* = 3 uc) serves as a tunneling barrier with the major defects deliberately suppressed; the V_O_ defects which serve as donors are solely confined in the top *n* = 2 or 8 uc LAO layer through rapid annealing (RA) (see sample growth and characterization and Fig. S1).

Spectra from sum frequency generation (SFG) (Fig. 1(b)), a nonlinear optical process that is very sensitive to interfacial Ti-O bonds, show that the characteristic phonon mode for V_O_s (96-meV) is suppressed for (3 + 2) LAO/STO samples (red curve) while remaining prominent for traditionally grown (5 + 0) samples [23]. This result suggests the interface is virtually free of V_O_s in a (3 + *n*) sample. Additional annular dark-field scanning transmission electron microscopy (ADF-STEM) shows that ion intermixing is also minimal (see sample growth and characterization and Fig. S2).

As a result, the average low-field mobilities for as-grown (3 + *n*) LAO/STO samples can easily reach >10 000 cm^2^/Vs, which is significantly higher than the traditional continuously grown (5 + 0) samples and (3 + 2) samples without RA (Fig. 1(c)). Further multiband analysis reveals that the mobilities of high mobility subband of (3 + 2) samples can reach >46 000 cm^2^/Vs.

## RESULTS

### Anomalous quantum oscillations

The quantum oscillations at the LAO/STO interface are rather complicated compared to simple 2DEGs on semiconductor heterostructures and 2D materials. The main discrepancy lies in inconsistent reports on the aperiodicity, assignment of the orbitals, and band degeneracies and crossings [24]. By studying nanowires fabricated by conductive atomic force microscope (cAFM), we previously proposed that channeled high mobility transport on FDW could reconcile all the known discrepancies at the 2D LAO/STO interface [25]. Specifically, the lateral confinement on quasi-1D FDW imposes additional energy on top of Landau quantization, which makes the energy ladder aperiodic (in the 1/*B* axis) [26]. However, the reason why the carriers on FDWs and cAFM nanowires (FDW by nature) have enhanced mobilities and even perfect quantum ballistic transport remains a puzzle.

Fig. 1(d) shows the normalized sheet resistances $\frac{{{R_{\mathrm{s}}}( B )}}{{{R_{\mathrm{s}}}( 0 )}}$ of three (3 + *n*) LAO/STO samples (labeled as A, B, and C) at high back gate voltages ${V_{{\mathrm{bg}}}}$ (∼200 V). All the measurements were taken at *T* = 1.5 K in the van der Pauw geometry with 5 $mm \times $ 5 $mm$ sample sizes. Although the profiles of magnetoresistance vary, they all show pronounced oscillations persistent to low magnetic fields ∼0.3 T, especially for thicker (3 + 8) samples. The second derivative of sheet resistance $- \frac{{{{\mathrm{d}}^2}{R_{\mathrm{s}}}}}{{{\mathrm{d}}{B^2}}}$ reveals several anomalous features (Fig. 1(e)). First, these oscillations are all aperiodic on the 1/*B* axis. Second, they appear primarily on low magnetic fields below 2 T. Third, the oscillation amplitudes rapidly decrease with increasing magnetic fields, in contrast to the standard Shubnikov–de Hass (SdH) oscillations [27]. Additionally, these oscillations are surprisingly reproducible in nearly all ∼60 (3 + *n*) samples grown.

The aperiodicity can be well explained by the magnetic depopulation effect on the quasi-1D FDWs. We analyze the oscillation in an extended Lifshitz–Kosevic (LK) framework by including geometric confinement [27]. Namely, the effective oscillation frequency is given by $\Omega = \sqrt {\omega _c^2 + \omega _y^2} $, where ${\omega _c} = eB/{m_0}$ and ${\omega _y}$ are cyclotron frequency and lateral confinement frequency of FDWs, respectively; ${m_0}$ is the cyclotron effective mass. As shown in Fig. 1(f), the positions of peaks and valleys in the oscillations can be fitted with a generalized Lifshitz–Onsager quantization rule with confinement renormalized to $\Omega $ and a dimensional phase shift,


(1) \begin{eqnarray*}
n = \frac{{{\Omega _F}}}{\Omega } - \delta + {\mathrm{\gamma }},
\end{eqnarray*}

where *n* is the index of the magnetoelectric subband, $1/{\Omega _F}$ is the frequency for oscillations with respect to $1/\Omega $, $\gamma = \frac{1}{2} - \frac{\phi }{{2\pi }}$, $\phi $ is the Berry phase, and $\delta = 0{\mathrm{\,\,}}$($\pm \frac{1}{8})$ is the phase shift for 2D (3D and 1D) dimensionality [28] (see Supplementary Data). The relation yields highly nonlinear $n{\sim} \frac{1}{B}$ curves in the low magnetic fields, as opposed to the linear dependence of the Landau fan diagram in 2DEGs. This nonlinearity can be understood as a quasi-1D to 3D transition since ${\omega _y}$ is dominant only at low fields, while at high fields (${\omega _c} \gg {\omega _y}$) the quasi-1D magnetoelectric subbands turn to regular Landau orbitals. We note the extraction of the Berry phase is unreliable due to the high sensitivity on the high field peak and valley positions, which have large uncertainties due to diminishing oscillation amplitudes.

### Angle dependence

**Figure 2.**
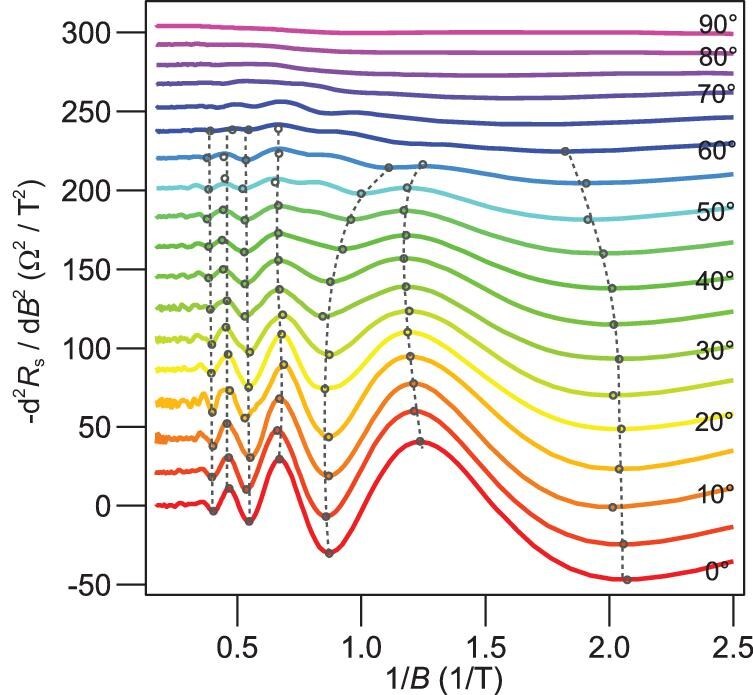
Angular dependence of the anomalous quantum oscillations. The second derivative of sheet resistance $- \frac{{{{\rm d}^2}{R_s}}}{{{\rm d}{B^2}}}$ as a function of magnetic field angles $\theta $ ranging from ${0^\circ }$ to ${90^\circ }$. $\theta $ is the angle between the magnetic field direction and the [001] plane, as depicted in the schematic structure in Fig. 1(a). The data has been manually shifted for clarity. The dashed lines mark the evolution of oscillations with tilt angles, indicating the quasi-1D and 3D dimensionality at the low and high magnetic fields, respectively.

To further elucidate the dimensionality, we study the angle dependence of the anomalous quantum oscillation by rotating samples with respect to the magnetic field direction. As shown in Fig. 1(a) and Fig. 2, the oscillation amplitude gradually decreases as the field direction relative to the sample changes from out-of-plane ($\theta = 0^\circ $) to in-plane ($\theta = 90^\circ $). The peak and valley positions slightly shift at low magnetic fields while staying constant at high fields. This is consistent with quasi-1D to quasi-3D transition illustrated in the nonlinear Landau fan diagram (Fig. 1(f)). As the magnetic length ${d_c} = 2\sqrt {\frac{{\hbar ( {2n + 1} )}}{{m_e^{\mathrm{*}}{\omega _c}}}} $ becomes smaller compared to the effective channel width $W = l_y^2\sqrt {\frac{{2m_e^{\mathrm{*}}{{\mathrm{\Omega }}_F}}}{\hbar }} $ on FDWs with increasing magnetic field, the quantum oscillations become increasingly 3D like in the high magnetic fields.

The angle dependence can rule out the possibility of *B*-periodic Sondheimer oscillations, whose oscillation period is inversely proportional to the thickness of the quasi-2DEG and should change with the sample rotation angle [29]. We also note that alternate explanations of the aperiodicity of quantum oscillations exist [30], which involve corrections from high-order magnetic response functions through the Gao-Niu quantization condition [31], or nonideal Dirac bands [32]. However, these scenarios mainly work for strong magnetic fields where high-order effects are nonnegligible, in contrast to the low-field aperiodic oscillation observed in this work. Finally, we emphasize that observing these oscillations does not necessitate sample-wide coherence; instead, it relies on uniform lateral confinement throughout the sample.

### Effective mass

**Figure 3.**
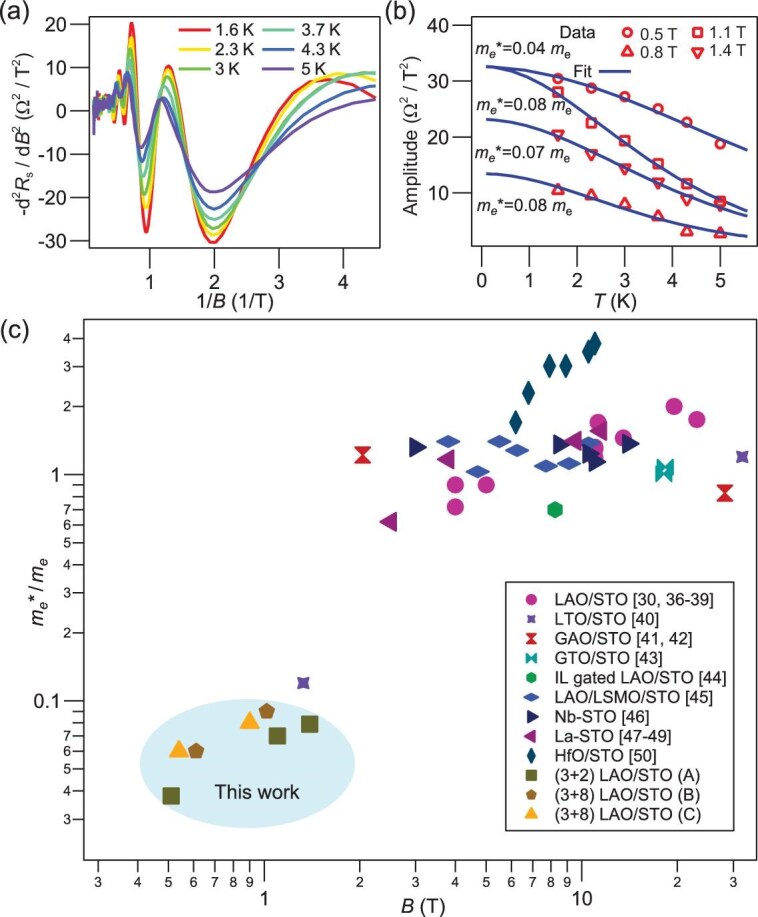
Effective mass. (a) Temperature-dependent anomalous quantum oscillations of sample A. (b) Temperature-dependent oscillation amplitude at different magnetic fields. Fitting to LK relation reveals the effective mass can be as small as 0.04m_e_. (c) Survey of effective masses of STO-based electron systems, which reveals unprecedently light electrons in this work.

The emergence of quantum oscillations at ultra-low magnetic fields suggests the associated carriers are very light, reminiscent of massless Dirac particles in clean graphene [33] and topological insulators [34], and light subbands in GaAs/AlGaAs quantum wells [35]. The effective mass can be measured from the temperature-dependent oscillation amplitudes in the LK equation $\frac{{{{\mathrm{d}}^2}{R_s}}}{{d{B^2}}} \propto \frac{{\beta T}}{{\sinh ( {\beta T} )}}$, where $\beta = \frac{{2{\pi ^2}{k_B}}}{{\hbar \Omega }}$. This equation essentially describes the oscillation amplitudes decay with increasing temperature due to the thermal smearing of the electron distribution around the Fermi level, which is still valid for the extended LK model here (see Supplementary Data for details). Figure 3 shows the fitting of sample A data to the LK equation for oscillations for temperatures ranging from 1.6 K to 5 K. The extracted effective masses ($0.04{m_e}\sim0.08{m_e})$ at different magnetic fields are indeed extremely small, where ${m_e}\,\,$is the bare electron mass.

Such a light subband is very surprising, provided the electronic structure of the LAO/STO interface has been extensively studied over the last two decades. In fact, as a prime platform for studying correlated physics, the effective mass of Ti *t*_2 g_ orbitals in STO-based electron systems is usually heavy ($0.6{m_e} < m_e^* < 4{m_e})$ [30,36–50], as shown in the survey of effective masses in literature in Fig. 3(c). The only exception is the ultra-thin LaTiO_3_/STO (LTO/STO) interface reported by Veit *et al*., where carriers with an effective mass of $0.12{m_e}$ were reported [40]. They proposed a phenomenological model by including a giant linear Rashba spin-orbit coupling (SOC) term with a Rashba parameter ${\alpha _R} = 1.8\,\, \times {10^{ - 11}}\,\,{\mathrm{eV\,\,m}}$, which significantly reduces the mass of inner hybridized ${d_{xz + yz}}$orbitals. This scenario appears applicable to our work; however, ${\alpha _R}$ at the LAO/STO interface is typically one order smaller ($3.4\,\, \times {10^{ - 12}}\,\,{\mathrm{eV\,\,m}}$) [51], which is unlikely to support even lighter subbands.

### TRS-protected transport

Considering the extensively studied 2D LAO/STO interface, the sole conceivable place for hosting such light carriers are the much less explored FDWs. It is well known that the band structure of the domain walls can be modified from the bulk materials due to local symmetry breaking in forms of band gap reduction, band bending, and metal-insulator transitions [52], as has been observed in materials including BiFeO_3_ [53] and Nd_2_Ir_2_O_7_ [54]. The FDWs in STO are polar due to local inversion symmetry breaking [55]. The associated polar field could, in principle, give rise to a much larger Rashba SOC than that of the interface, allowing novel electronic phases to emerge. For example, Yerzhakov *et al*. proposed that Majorana zero modes could appear on FDWs in STO by the interplay of strong Rashba SOC, ferroelectric polarization, and intrinsic superconductivity [55]. Fidkowski *et al*. proposed that the cAFM nanowire (FDW by nature) at the LAO/STO interface can be a ‘helical quantum wire,’ essentially an edge of a 2D quantum spin Hall insulator [56,57].

**Figure 4.**
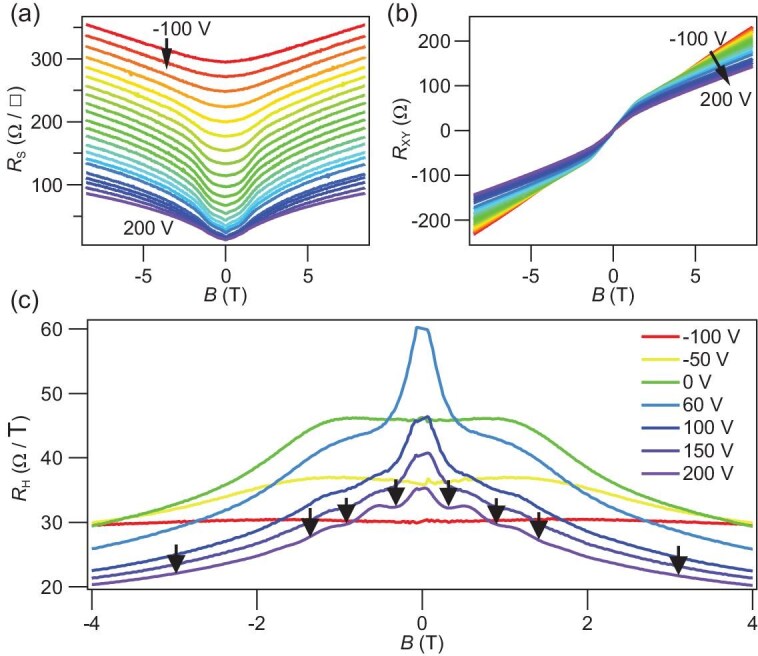
Gate-tunable transport of Sample A. (a) Sheet resistance ${R_{\mathrm{s}}}$ as ${V_{{\mathrm{bg}}}}$ sweeps from $- 100{\mathrm{\,\,V}}$ to $200{\mathrm{\,\,V}}$. ${R_{\mathrm{s}}}{\mathrm{\,\,}}$evolves from a simple parabolic shape to a more complex dependence on the magnetic field. (b) Simultaneously measured ${R_{{\mathrm{xy}}}}$ curves become nonlinear with a characteristic ‘S’ shape, suggesting multiband transport. (c) Hall coefficient ${R_{\mathrm{H}}}{\mathrm{\,\,}}$at different back gate voltages $- 100\,\,{\mathrm{V}} < {V_{{\mathrm{bg}}}} < 200\,\,{\mathrm{V}}$. The profile of ${R_{\mathrm{H}}}$ turns from constant to a bell shape ($- 4\,\,{\mathrm{T}} < B < 4\,\,{\mathrm{T}}$), with a dip-to-peak transition in low fields ($- 1.5\,\,{\mathrm{T}} < B < 1.5\,\,{\mathrm{T}}$) at ${V_{{\mathrm{bg}}}} > - 50\,\,{\mathrm{V}}$. The black arrows mark the quantum oscillations at ${V_{{\mathrm{bg}}}} = 200\,\,{\mathrm{V}}$.

We find all the transport signatures are consistent with a linear quasi-1D Rashba model on FDWs. Although not as robust as topological protection, TRS protection is present due to the strong Rashba SOC on FDWs that enforces spin and momentum locking in quasi-1D and suppresses backscattering at low fields (see Supplementary Data and Fig. S6) [58]. The presence of *k*-linear Rashba SOC ${H_{{\mathrm{SO}}}} = {\alpha _R}( {\hat n \times \vec k} ) \cdot \vec \sigma $ leads to two energy bands ${E_ \pm }( k ) = \frac{{{\hbar ^2}{k^2}}}{{2{m_0}}} \pm {\alpha _R}| k |$ and creates two Fermi pockets, where $\hat n$ is the unit vector in the polarity direction, $\vec \sigma $ is the Pauli matrix and ${m_0} = 0.6{m_e}$ [49] is the lower limit of cyclotron mass of light bands experimentally observed in STO-based systems (Fig. 3). This scenario will predominantly occur in FDWs between *Z* and *X* (*Y*) domains, where $\hat n$ is perpendicular to $\vec k$ and *X, Y* and *Z* denote the tetragonal direction along crystal axes [15,55]. In the 2D/3D limit where a circular inner Fermi pocket can be approximated, we can estimate ${\alpha _R}$ using


(2) \begin{eqnarray*}
m_e^{\mathrm{*}} = {m_0}\left( {1 - \frac{{{\alpha _R}}}{{\sqrt {\alpha _R^2 + 2{{\mathrm{\Omega }}_F}{\hbar ^3}/{m_0}} }}} \right).
\end{eqnarray*}

For $m_e^* = 0.08{m_e}$ at $B = 1.4\,\,{\mathrm{T}}$ of sample A, a Rashba parameter ${\alpha _R} = 1.1\,\, \times {10^{ - 10}}\,\,{\mathrm{eV\,\,m}}$ can be extracted. This value is on the same order of topological insulator Bi_2_Se_3_ ($4.0\,\, \times {10^{ - 10}}\,\,{\mathrm{eV\,\,m}})$ [59] and polar semiconductor BiTeI ($3.8\,\, \times {10^{ - 10}}\,\,{\mathrm{eV\,\,m}}$) [60], while being two orders larger than the LAO/STO 2D interface [14].

Generally, the amplitude of SdH oscillations increases with the magnetic field as the spacing between Landau levels widens. However, the observed low-field oscillations, both in resistance and conductance, surprisingly behave oppositely (see Fig. S5). This phenomenon can be understood as transport being protected by TRS at zero field in a quasi-1D Rashba wire. No backscattering channel is available since spins and momenta are tightly locked. Applying a magnetic field mixes spins and opens backscattering channels, eventually suppressing quantum oscillations by Anderson localization. This TRS-breaking process is commonly observed in the helical edge state of quantum spin Hall insulators in small magnetic fields [57,61]. In the meantime, high-field (up to 14 T) quantum oscillations are absent in most samples, possibly due to reduced mobilities at high fields (see Fig. S11) after quenching the TRS-protected subband and enhanced mass for the outer Fermi pocket.

### Gate-tunable TRS-protected subband

Next, we show that the TRS-protected subband is gate-tunable and can be described by a multiband model with quantum corrections. Unlike semiconductor heterostructures, where electrical gating primarily adjusts the Fermi level, back gating at the LAO/STO interface mainly tunes the disorder and superconductivity [62,63]. Upon back gating ($- 100\,\,{\mathrm{V}} < {V_{{\mathrm{bg}}}} < 200\,\,{\mathrm{V}})$ at $T = 1.5\,\,{\mathrm{K}},$ the sheet resistance ${R_s}\,\,$of sample A evolves from a simple parabolic shape without the quantum oscillations at ${V_{{\mathrm{bg}}}} = - 100\,\,{\mathrm{V}}$ to a more complex dependence on the magnetic field at ${V_{{\mathrm{bg}}}} > 0{\mathrm{\,\,V}}$ (Fig. 4(a)). Correspondingly, Hall resistance ${R_{{\mathrm{xy}}}}$ curves are relatively linear, and the Hall coefficient ${R_{\mathrm{H}}} = \frac{{{R_{{\mathrm{xy}}}}}}{B}$ stays constant at ${V_{{\mathrm{bg}}}} = - 100{\mathrm{\,\,V}}$ (Fig. 4). With increasing ${V_{{\mathrm{bg}}}}$, ${R_{{\mathrm{xy}}}}$ curves become nonlinear with a characteristic ‘*S*’ shape (Fig. 4(b)) and ${R_{\mathrm{H}}}$ curves simultaneously develop a bell shape with fine features at low magnetic fields (Fig. 4(a), 4(b)). Two observations in ${R_{\mathrm{H}}}$ are evident: (1) the ${R_{\mathrm{H}}}$ curve initially dips over a bell shape background (${V_{{\mathrm{bg}}}} < 0\,\,{\mathrm{V}})$, then flattens out at ${V_{{\mathrm{bg}}}} = 0\,\,{\mathrm{V}}$ and peaks sharply at ${V_{{\mathrm{bg}}}} > 0\,\,{\mathrm{V}}$ at low magnetic fields ($| B | < 1.5{\mathrm{\,\,T}}$); (2) staircase features in ${R_{\mathrm{H}}}{\mathrm{\,\,}}$emerge at high ${V_{{\mathrm{bg}}}}$ values ($> \sim100\,\,{\mathrm{V}}$) and low magnetic fields, which are clearly related to the quantum oscillations.

The nonlinear Hall effect at the LAO/STO interface has been predominantly explained by the two-band model in the literature, in which the characteristic ‘*S*’ shape in$\,\,{R_{{\mathrm{xy}}}}$ and the bell shape in ${R_{\mathrm{H}}}\,\,$are caused by a second high-mobility electron band [64,65]. Although not routinely studied, the dip feature inside the bell shape in the ${R_{\mathrm{H}}}$ curve most naturally suggests the emergence of hole carriers. This possibility is immediately dropped in literature since the TiO_2_-terminated LAO/STO interface is *n*-type. However, with the presence of the Dirac point in the proposed linear Rashba model, this whole picture is still possible, and the dip-to-peak transition in ${R_{\mathrm{H}}}$ can be understood as the hole-to-electron transition. Alternatively, Gunkel *et al.* added an anomalous Hall effect (AHE) term originating from interfacial magnetism to the two-band model to explain the dip feature. This combined AHE model works well with NdGaO_3_/STO and defect-tuned LAO/STO interfaces, which are both magnetic and have low mobility [65,66]. While magnetism at LAO/STO interfaces is still highly controversial [67], our ultra-clean LAO/STO samples are presumably non-magnetic due to the absence of interfacial oxygen vacancies. In addition, no magnetic hysteresis is observed in all ${R_{\mathrm{s}}}\,\,$and ${R_{{\mathrm{xy}}}}$ curves (see Supplementary Data and Fig. S8).

**Figure 5.**
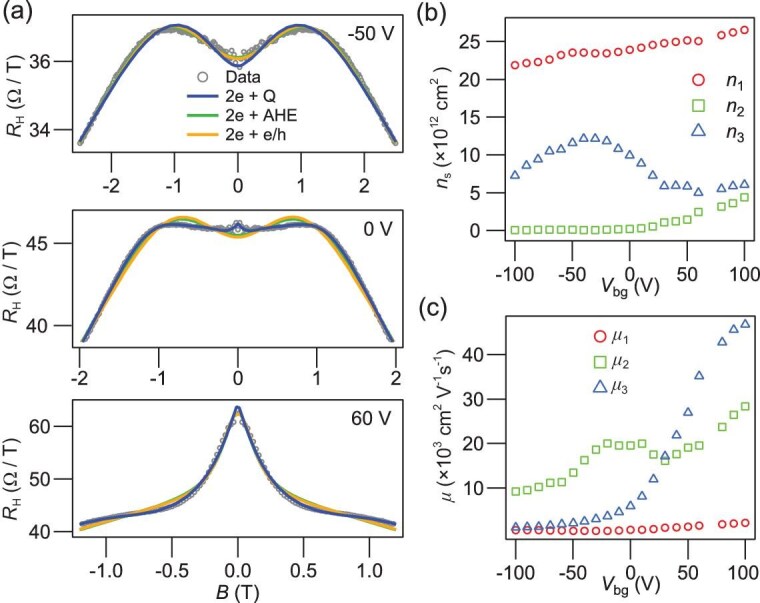
Quantum-corrected multiband fitting. (a) Fitting with various versions of the multiband models. It is obvious that the quantum-corrected multiband model yields the best fitting. 2e + Q: quantum corrected multiband model; 2e + AHE: two-band model with AHE; 2e + e/h: two-band model with electron to hole transition. Carrier densities (b) and mobilities (c) of the three subbands at B = 0 T, respectively, extracted from the 2e + Q model.

Nevertheless, we check the applicability of the whole picture and AHE picture by fitting the transport data with a three-band model, which includes an additional band to reflect hole-to-electron transition or AHE contribution on the basis of the classical two-band model. As a result, neither model can faithfully fit the data across the dip-to-peak transition in ${R_{\mathrm{H}}}$ (Fig. 5(a)) and yields unrealistic fitting parameters, as is evident in the goodness of fit using the root-mean-square-error (RMSE) (see Figs S9 and S10). Furthermore, our gating data does not fall under the universal scaling law at LAO/STO interfaces observed by Joshua *et al*. [64], which states that a critical carrier density (∼$1.6{\mathrm{\,\,}} \times {10^{13}}{\mathrm{\,\,c}}{{\mathrm{m}}^{ - 2}})$ exists at the Lifshitz transition from ${d_{xy}}$ to hybrid ${d_{xz + yz}}$ bands (see Fig. S12). These deviations suggest that our ultra-clean samples cannot be described by the classical multiband model with a rigid band structure.

All the features in the back-gating data are consistent with the proposed quasi-1D Rashba model and multiband transport with quantum corrections. In the classical multiband model, carrier densities and mobilities are independent of the magnetic field. However, the TRS-protected transport on FDWs is clearly field-dependent. It manifests itself as the mobility of the associated subband sharply decreases in the TRS-breaking process caused by the magnetic field. We rewrite the three-band model to


(3) \begin{eqnarray*}
{R_H} = - \frac{1}{e}\frac{{\mathop \sum \nolimits_j \frac{{{n_j}\mu _j^2}}{{1 + \mu _j^2{B^2}}}}}{{{{\left( {\mathop \sum \nolimits_j \frac{{{n_j}{\mu _j}}}{{1 + \mu _j^2{B^2}}}} \right)}^2} + {{\left( {\mathop \sum \nolimits_j \frac{{{n_j}\mu _j^2}}{{1 + \mu _j^2{B^2}}}} \right)}^2}{B^2}}},
\end{eqnarray*}

where ${n_j}$ and ${\mu _j}\,\,( {j = 1,2,3} )$ are the carrier density and mobility of each subband, ${\mu _3}( B ) = \mu _3^0\exp ( { - \frac{B}{{{B_c}}}} )$ is the mobility of the subband on the FDWs corrected by an exponential dependence of magnetic field, with $\mu _3^0$ the zero-field mobility and ${B_{\mathrm{c}}}$ the characteristic field (see Supplementary Data). This model provides an excellent fit to the data by capturing all the details of the dip-to-peak transition. Fig. 5(b) and 5(c) shows the extracted ${n_j}$ and ${\mu _j}$, which reveal a quantum-corrected subband (${n_3}$,$\,\,{\mu _3}$) with negative electronic compressibility and rapid onset of mobility from ∼6000 cm^2^/Vs (${V_{{\mathrm{bg}}}} = 0\,{\mathrm{V}})\,\,$to over 46 000 cm^2^/Vs (${V_{{\mathrm{bg}}}} = 100\,{\mathrm{V}})$.

## DISCUSSION

All the experimental evidence, including the aperiodic quantum oscillations at the ultra-low magnetic fields, extremely small effective mass, suppression of oscillation at high magnetic fields, and quantum-corrected multiband transport, are consistent with the TRS-protected transport on FDWs with a giant Rashba SOC. Under this framework, we next discuss possible insights into the complex LAO/STO interface.

We can understand why this TRS-protected state has not been explicitly observed over the past two decades. It is well-known that FDWs are prone to trap oxygen vacancies [19], which compensate the polar field [20] and subsequently reduce the Rashba SOC. In addition, oxygen vacancies increase disorder, which easily causes backscattering in the quasi-1D dimension. The oxygen vacancies are intentionally suppressed in our ultra-clean LAO/STO samples, as revealed by SF spectra and transport measurements. This could revive the strong Rashba SOC on FDWs and lead to the observed anomalous quantum oscillations.

A related question is whether the weak antilocalization (WAL) effect is relevant, which manifests as a sharp dip in low field *R*_s_ and is frequently used to study interfacial Rashba SOC [14]. There are no definitive signatures of WAL in the four samples reported here, they are either nonexistent or too small compared to the quantum-corrected multiband transport (Fig. 4). We point out that WAL is a 2D effect that should be suppressed in quasi-1D FDWs due to the absence of interfering scattering paths.

The strongly ionic nature of the complex oxide interface leads to an intricate set of subbands displaced vertically in real space. In principle, including more bands in the multiband model will describe the interface more precisely [68]; however, the three-band model can at least qualitatively tell the subband characteristics. It is plausible to recognize the quantum-corrected band (${n_3}$,$\,\,{\mu _3}$) as the same subband of anomalous quantum oscillations due to their inherent sensitivity to the magnetic field and back gating. Namely, back gating tunes disorder level and Rashba SOC strength and gives rise to the enhanced mobility and anomalous quantum oscillation at high ${V_{{\mathrm{bg}}}}$. Meanwhile, subband (${n_1}$,$\,\,{\mu _1}$) with high density and low mobility can be attributed to carriers close to the interface or the outer Fermi pocket on FDWs, both with a larger mass and higher density. Subband (${n_2}$,$\,\,{\mu _2}$) likely originates from high mobility interfacial electrons, which quickly populate at ${V_{{\mathrm{bg}}}} > \sim30\,\,{\mathrm{V}}$.

Finally, it is intriguing to know the relationship between superconductivity and anomalous quantum oscillations for a correlated interface. Superconductivity in STO is often related to the ferroelectric mode [69,70], with electron pairing mediated by polar instability or ferroelastic waves on FDWs [71]. Indeed, superconducting *T*_c_ is found to be higher on FDWs [18]. We cool down samples A to mK temperatures and extract a phase diagram as a function of temperature and ${V_{{\mathrm{bg}}}}.$ Interestingly, superconductivity persists up to the ${V_{{\mathrm{bg}}}}$ values at which anomalous quantum oscillations start to appear (within ∼50 V due to sample inhomogeneity and gate uncertainty), as shown in Figs S13 and S14. This coincidence suggests the possible existence of a quantum critical point that marks the transition between superconductivity and quantum oscillations. Moreover, while ${n_1}$ is mostly constant and ${n_2}$ is not large enough to support superconductivity in the range of $- 40\,\,{\mathrm{V}} < {V_{{\mathrm{bg}}}} < 50\,\,{\mathrm{V}}$, the carrier density ${n_3}$ of the quantum-corrected band (${n_3}$,$\,\,{\mu _3}$) varies significantly (>50%), which matches the profile of the superconducting dome. This observation points to a direct connection between superconductivity and FDWs.

## CONCLUSIONS

In summary, our experiments uncover an anomalous light subband at the correlated LAO/STO interfaces. All the experimental evidence points to a TRS-protected state on quasi-1D FDWs, with a Rashba SOC strength in the same order as typical topological insulators. Our work adds the missing TRS-protected physics to the rich set of emergent phenomena, paving the way to quantum engineering of novel states of matter at the oxide interfaces.

## Supplementary Material

nwaf156_Supplemental_Files
