# Supplementary material for: Time reversal symmetry-protected transport at correlated oxide interfaces
Source: Natl Sci Rev. 2025 May 7;12(6):nwaf156. doi: 10.1093/nsr/nwaf156 (PMC12163994; doi:10.1093/nsr/nwaf156)
Supplement: nwaf156_Supplemental_Files [file nwaf156_supplemental_files.zip › Supplementary data.docx]

Supplementary Data for

Time-Reversal Symmetry Protected Subband at Correlated Oxide Interfaces

Mengke Ha *et al.*

*Corresponding author: Jiangfeng Du, [djf@ustc.edu.cn](mailto:djf@ustc.edu.cn); Guanglei Cheng, [glcheng@ustc.edu](mailto:glcheng@ustc.edu).cn.

**This PDF file includes:**

Supplementary Text

Figs. S1 to S14

Tables S1 to S2

References (*1-9*)

Supplementary Text

SAMPLE GROWTH AND CHARACTERIZATION

1. Sample growth

LAO/STO samples were epitaxially grown by pulsed laser deposition (PLD) equipped with a reflection high-energy electron diffraction (RHEED) system. Before growth, (100) STO substrates were treated by buffered HF and annealed in an oxygen environment at 950 $℃$ to achieve an atomically smooth surface. The laser spot has a flat-top profile, which is crucial to achieving the desired results. The laser fluence is 0.9 J/cm^2^ with 1 Hz repetition frequency. The sample growth was broken into five stages (Fig. S1):

1. Pre-annealing. STO substrate is heated up to 650 $℃$ by a laser in $P_{O_{2}}=100$Torr to remove contaminants at the surface.
2. Interfacial LAO growth. The interfacial buffer LAO layer (*m*=3 uc) is deposited at 550 $℃$ with a low oxygen partial pressure $P_{O_{2}}=5\times{10}^{-6}$Torr to introduce abundant V_O_s, which compensates for the built-in potential $\phi_{LAO}$ arising from polar discontinuity and reduce ion intermixing (driven by $\phi_{LAO}$) and V_Sr_ formation at the interface.
3. Intermediate annealing. These interfacial V_O_s are subsequently removed by annealing in high $P_{O_{2}}=200$ Torr (for 1~3 hours) at a lower temperature *T*=430 $℃$, at which ion intermixing is still mitigated due to much lower diffusion coefficients of metal ions than that of oxygen ions. This procedure creates virtually defect-free LAO/STO interfaces.
4. Top LAO growth. The top *n*=2 or 5 uc LAO layer is then grown at the annealing temperature (430 $℃$) with a low $P_{O_{2}}=5\times{10}^{-6}$Torr to generate V_O_ donors, which transfer electrons to the interface. RHEED oscillations are observed in both procedures (Fig. S1). Such (*m*+*n*) configuration essentially enables a modulation doping charge transfer process.
5. Rapid annealing (RA). Finally, the (*m*+*n*) LAO/STO sample is rapidly cooled to room temperature within 30 seconds in an oxygen environment (tuned to several Torr to control the carrier densities) once the laser is turned off and is immediately transferred to the nitrogen-filled load lock to provide further cooling. This RA procedure is vital to prevent the migration of V_O_s to the interface and degrade the interface quality.

The (5+0) samples were continuously grown at the same growth conditions, except *m*=5 and without the top LAO growth stage. This is the conventional method for growing LAO/STO samples, which serve as control samples in this work.

1. Sum Frequency Generation (SFG) Spectroscopy

We utilize SFG phonon spectroscopy to probe the interfacial oxygen vacancies in (3+2) uc and (5+0) uc LAO/STO samples[1]. Broadband infrared and narrowband pulses, generated by a 35-fs pulsed laser amplifier (Spitfire ACE, Spectra Physics) of 800 nm wavelength at 2 kHz repetition rate, are overlapped at the sample surface after pumping an optical parameter amplifier followed by a difference frequency generation stage (TOPAS-C, Spectra-Physics). The resultant SFG signal is very sensitive to inversion symmetry breaking, an ideal tool for probing interfacial phonons in STO. We previously reported that the 96-meV resonance peak is characteristic of V_O_s at the interface. This peak is suppressed in (3+2) uc samples, indicating V_O_ is significantly less in these samples than in continuously grown (5+0) samples.

1. ADF-STEM

ADF-STEM is performed by the Grand ARM 300F (JEOL) with an aberration corrector operated at 300 kV. As shown in Fig. S2, the high-resolution mapping image shows the atomically abrupt interface of (3+2) uc LAO/STO interface, with no sign of ion intermixing. The detailed structure of the top 2 uc is less sharp, suggesting the deficiency of oxygen atoms. We also detect oxygen deficiency along the top atomic plane and V_O_s are obtained expectedly. These characterizations suggest the modulation doping mechanism in (3+2) uc LAO/STO heterostructure.

ENERGY DIAGRAM and Calculation details

Density functional theory (DFT) calculations were used to analyze the defect formation in the LAO layer in such a growth strategy. The formation energy $\Delta H\left( \varepsilon_{F}\left( x \right), X^{q} \right)$ of defect $X^{q}$ in LAO depends on the distance $x$ between the defect and the LAO/STO interface, since the Fermi level relative to local valence band maximum (VBM) $\varepsilon_{F}\left( x \right)$ is different due to the polarization electric field within LAO. As shown in Fig. S3(a), in O_2_ poor environment, the $\varepsilon_{F}(3 uc)$ is ~1.25 eV and the formation energy of V_O_s is close to zero. The formation of antisite defects $\mathrm{Ti}_{\mathrm{Al}}^{+}$is also suppressed in O_2_ poor environment, so the major defect in LAO is V_O_ during the first interfacial 3 uc LAO growth, consistent with the previous calculations [2]. The migration energy of $\mathrm{Ti}^{4+}$ (~8.3 eV) is much higher than O^2-^ (~0.5 eV) and is still high (~4.2 eV) with the assistance of V_Sr_, so the annealing at low temperature and rich O_2_ atmosphere can effectively eliminate V_O_s without activating ion intermixing at the interface. In addition, the migration of $\mathrm{Sr}^{2+}$ is also slow with ~3.7 eV migration energy and contributes less compensating field. In the first 3 uc LAO after annealing, the electric field remains high, and the Fermi level $E_{F}$ is slightly lower than the conduction band minimum (CBM) (Fig. S3(b)).

Finally, the fast quenching of the top 2 uc LAO layers makes them rich in intrinsic defects, such as V_La_, V_Al_ and V_O_, which will compensate the field as shown in Fig. S3(b). Meanwhile, their relative amount can be tuned by growth oxygen partial pressure. In O_2_ poor environment, more V_O_ defects will be formed, which donate more electrons to the CBM of STO to form 2DEG. Therefore, a clean interface is expected with such a sample growth strategy.

All calculations were performed using DFT and the plane-wave projector-augmented wave method as implemented in the VASP code. An energy cutoff of 520 eV and the PBEsol exchange correlation functional was adopted to relax the structures. HSE06 hybrid functional was used to calculate the energy on top of these relaxed structures for better describing the defect level and band gap in the formation energy calculation. The Brillouin zone was sampled by $6\times6\times6$ and $1\times1\times1$ *k*-point mesh centered on $\Gamma$ point for primitive cell of 5 atoms and supercell of 135 atoms, respectively. The atomic forces were relaxed to be less than 0.01 eV/Å. The calculated lattice parameters of STO and LAO are 3.895 Å and 3.771 Å.

TRANSPORT MEASUREMENT

We use reverse-field reciprocity to avoid sweeping the field, which is applicable as far as the sample obeys the Onsager reciprocal relation (i.e. ohmic), and does not show magnetic hysteresis [3]. We also adopt a spinning current Hall measurement method in a van der Pauw (vdP) geometry to achieve fast and precise measurement. The method is frequently used in commercial Hall sensors to achieve nanotesla sensitivity [4].

Specifically, 4 aluminum wires are punched at corners to contact the interface by wire bonding in typical $5 mm\times5 mm$ samples, as shown in Fig. S4. Eight measurement configurations, labeled from A to H in Table S1, are quickly permutated in a small magnetic field (*B*=0.5 T) using a matrix switch. In each configuration, an *I*-*V* curve is measured to extract the resistance $R_{ij,mn}$, which represents the resistance measured by sourcing current $I_{ij}$ from contact *i* to *j*, and measuring the voltage difference $V_{mn}$ between contact *m* and *n*. The linearity of each *I*-*V* curve is also used as a sanity check for the quality of ohmic contact of wire bonds at any conditions, which we found extremely important in correctly interpreting experiment data.

For longitudinal configurations A to D, the common false signals in longitudinal voltage measurement are thermoelectric voltage $V_{th}^{mn}$ originating from the temperature difference between contacts *m* and *n*, and misalignment voltage originating from the crosstalk between Hall voltage and longitudinal voltage due to geometrical misalignment of the contacts. The thermoelectric voltage can be easily removed by differentiating the *I*-*V* curve since it is mostly constant in a single *I*-*V* curve measurement. As a result, $R_{12,43}$ for configuration A can be written as

$$\begin{aligned} R_{12,43}=\frac{{dV}_{43}}{dI_{12}}=R_{a}+c_{1}R_{H}B，\#(S1) \end{aligned}$$

where $R_{a}$ is one of the two resistance values used in the van der Pauw formula, $R_{H}$ is the Hall coefficient, $B$ is the magnetic flux density and $c_{1}$ is a small constant due to contact misalignment.

According to reverse-field reciprocity, interchanging the current and voltage measurements (i.e., from configuration A to configuration C) effectively flips the magnetic field. Then we have

$$\begin{aligned} R_{34,21}=\frac{{dV}_{21}}{dI_{34}}=R_{a}-c_{1}R_{H}B，\#(S2) \end{aligned}$$

Averaging these two resistance values yields

$$\begin{aligned} R_{a}=\frac{1}{2}\left( R_{12,43}+R_{34,21} \right)，\#(S3)\#\#\#\#\# \end{aligned}$$

Similarly, we have the other resistance value $R_{b}$ in van der Pauw measurement

$$\begin{aligned} R_{b}=\frac{1}{2}\left( R_{23,14}+R_{41,32} \right)，\#(S4) \end{aligned}$$

The sheet resistance $R_{s}$ is then calculated by solving the van der Pauw equation

$$\begin{aligned} \exp\left( -\frac{\pi R_{a}}{R_{s}} \right)+\exp\left( -\frac{\pi R_{b}}{R_{s}} \right)=1，\#\left( S4 \right) \end{aligned}$$

Meanwhile, configurations E to H are applied to measure the Hall coefficient. The contribution of longitudinal voltage due to the misalignment of contact leads is eliminated by reverse-field reciprocity and averaging the four values:

$$\begin{aligned} R_{H}B= \frac{1}{4}\left( R_{13,42}+R_{24,13}+R_{13,42}+R_{13,42} \right)，\#\left( S5 \right) \end{aligned}$$

The carrier density $n_{e}$ and low-field Hall mobility $\mu_{H}$ are then given by

$$\begin{aligned} n_{e}=\frac{1}{eR_{H}},\#\left( S6 \right) \end{aligned}$$

$$\begin{aligned} \mu_{H}=\frac{1}{n_{e}eR_{s}},\#\left( S7 \right) \end{aligned}$$

where $e$ is the electron charge. All the configuration information is listed in Table S1.

LANDAU FAN, RASHBA MODEL AND REDUCED EFFECTIVE MASS

We plot the conductance of each sample as a function of 1/*B* with a smooth background removed (Fig. S5). The conductance oscillations show similar characteristics of resistance oscillations shown in Fig. 2(b), which are opposite to typical SdH oscillations.

To capture all these characteristics mentioned in the main text, we start from the Lifshitz–Onsager quantization in a typical high-mobility 2DEG,

$$\begin{aligned} n=\frac{hS_{F}}{4\pi^{2}eB}-\gamma,\#(S8)\# \end{aligned}$$

where $S_{F}$ is the extremal cross-sectional area of the Fermi surface, $\gamma=\frac{1}{2}-\frac{\phi}{2\pi},$ and $\phi$ is the Berry phase [5]. When it comes to a quasi-1D system where the effective width $W$ in real space is comparable to the magnetic length $d_{c}$, extra lateral electrostatic confinement needs to be considered in addition to the confinement caused by the applied perpendicular magnetic field. For free electrons, we have the Hamiltonian (See reference [6])

$$\begin{aligned} \hat{H}=\frac{\hat{p}_{y}^{2}}{2m^{*}}+\frac{m^{*}\Omega^{2}}{2}y^{2}+\frac{\hbar^{2}k_{x}^{2}}{2m^{*}},\#\left( S9 \right) \end{aligned}$$

where $\Omega={(\omega_{c}^{2}+\omega_{y}^{2})}^{\frac{1}{2}}$, $\omega_{y}=\frac{\hbar}{m_{0}l_{y}^{2}}$, $m^{*}$ is the effective cyclotron mass, $l_{y}$ is the characteristic width of the lateral confinement potential and $m_{0}$ is the cyclotron mass. Therefore, the quasi-1D nature renormalizes the strength of the magnetic field and adds an additional $k_{x}$ dependence in addition to the harmonic oscillator Hamiltonian. The latter is similar to the Landau level in 3D where an additional $k_{z}$ dependence also exists. So the corresponding energy spectrum $\epsilon_{n}$is given by

$$\begin{aligned} \epsilon_{n}\left( k_{x} \right)=\hbar\Omega\left( n+\frac{1}{2} \right)+\frac{\hbar^{2}k_{x}^{2}}{2m^{*}}\frac{\omega_{y}^{2}}{\Omega^{2}},\#\left( S10 \right) \end{aligned}$$

which is a series of vertically displaced parabolic magnetoelectric subbands and has a similar form to the 3D case.

For quasi-1D electrons with a general energy-momentum dependence, we adopt the same treatment as before, i.e., the confining potential modifies the magnetic field and an additional $k_{x}$

dependence is present. In that case, Eq. (S8) becomes

$$\begin{aligned} n=\frac{hS\left( \varepsilon,k_{x} \right)/m^{*}}{4\pi^{2}\Omega}-\gamma,\#\left( S11 \right) \end{aligned}$$

The resulting spectrum is therefore $\varepsilon=\varepsilon(n,k_{x})$.

Following the treatment of SdH oscillations in the LK framework in reference [7], we extend the analysis and find that

$$\begin{aligned} \rho=\rho_{0}[1+A\left( \Omega,T \right)\cos2\pi\left( \frac{\Omega_{F}}{\Omega}-\delta+\gamma\right),\#\left( S12 \right) \end{aligned}$$

where $A\left( \Omega,T \right)$=$\frac{\beta T}{sinh(\beta T)}e^{-{\beta T}_{D}}$, with$\beta={2\pi^{2}k_{B}}/{\hbar\Omega}$, $k_{B}$ is the Boltzmann constant, $T_{D}$ is Dingle temperature, $1/\Omega_{F}$ is the frequency for oscillations with respect to $1/\Omega$, and $\delta$ is an additional phase shift determined by the dimensionality, i.e., $\delta=0$ for the 2D case and $\delta=\pm1/8$ for the 3D or quasi-1D case. The positions of peaks and valleys in the oscillations can thus be described in Eq. (1) in the main text.

The energy spacing of a quasi-1D system in a magnetic field is described by $\hbar\Omega$, which no longer gives a linear relation between $n$ and $1/B$ in low fields as in 2D. With increasing magnetic fields, the energy spacing $\hbar\Omega$ $\to$ $\hbar\omega_{c}$ as Landau level spacing $\hbar\omega_{c}$ gradually dominates over the lateral electrostatic confinement. Meanwhile, the Landau level occupation turns to approximate linear in a pure 2D system, so $\gamma$ takes the formula of $\gamma=\frac{1}{2}-\frac{\phi}{2\pi}$.

In brief, the generalized Lifshitz–Onsager quantization rule can be described as equation S11. By analyzing the $n\sim1/B$ curves (Landau fan diagram) of the quantum oscillations, we can obtain values of $\Omega_{F}$, $l_{y}$, $d_{c}$ and $W$ of sample A~C listed in Table S2.

The inversion symmetry breaking and the polarity on FDWs suggest the possibility of a large Rashba SOC, characterized by a *k*-linear Rashba model

$$\begin{aligned} \hat{H}=\frac{\hbar^{2}k^{2}}{2m_{e}^{*}}\pm\alpha_{R}\left| k \right|,\#\left( S13 \right) \end{aligned}$$

As shown in Fig. S6, two Fermi pockets are emerging. In the 2D/3D limit for a fixed Fermi surface, the effective mass is given by

$$\begin{aligned} m_{e}^{*}=\frac{\hbar^{2}}{2\pi}\frac{\partial S_{F}}{\partial\epsilon_{F}},\#\left( S14 \right) \end{aligned}$$

where $S_{F}\sim\pi k^{2}$ is the area of the enclosed Fermi pocket, and the partial derivative is evaluated at the Fermi energy. In this case, we have the same explicit form for effective mass related to the inner fermi pocket that is responsible for low-field quantum oscillations

$$\begin{aligned} m_{e}^{*}=m_{0}\left( 1-\frac{\alpha_{R}}{\sqrt{{\alpha_{R}}^{2}+2\Omega_{F}{\hbar^{3}}/{m_{0}}}} \right),\#\left( S14 \right) \end{aligned}$$

At a given magnetic field, the temperature dependence of the oscillation amplitude can be described by the extended Lifshitz-Kosevich model: ${d^{2}R_{s}}/{dB^{2}}\sim{\beta T}/{sinh(\beta T)},$ with $\beta={2\pi^{2}k_{B}}/{\hbar\Omega}$. We fit the amplitudes of the second derivative of oscillations (from those we can reliably measure) to the model and show the results in Fig. S7 in addition to Fig. 4(b), which yields effective masses less than 0.1$m_{e}$. These exceptional light electrons are possibly arising from the inner Fermi surface produced from the bottom of the $d_{xy}$ band on FDWs.

MULTIBAND TRANSPORT

As $V_{\mathrm{bg}}$ sweeps from -100 V to 200 V, the sheet resistance $R_{s}$of sample A evolves from a simple parabolic shape to a more complex dependence on the magnetic field. Meanwhile, $R_{\mathrm{xy}}$ curves become nonlinear with a characteristic “*S*” shape (Fig. 4), suggesting multiband transport. Multiband transport is frequently applied to oxide interfaces where multiple electron orbitals are responsible for the transport. This model assumes that the mobilities of carriers do not change with the magnetic field and usually yields relatively good fitting for low mobility samples. However, it is clearly insufficient to describe the transport when additional processes are present, including the anomalous Hall effect (AHE) and quantum processes related to time-reversal symmetry (TRS). For the latter process, the mobility of the corresponding carrier will change as the TRS is broken by the magnetic field. Nevertheless, we discuss a comprehensive multiband model by combining the conventional multiband model and the contribution from AHE,

$$\begin{aligned} R_{H}=-\frac{1}{e}\frac{\sum_{j} \frac{n_{j}\mu_{j}^{2}}{1+\mu_{j}^{2}B^{2}}}{\left( \sum_{j} \frac{n_{j}\mu_{j}}{1+\mu_{j}^{2}B^{2}} \right)^{2}+\left( \sum_{j} \frac{n_{j}\mu_{j}^{2}}{1+\mu_{j}^{2}B^{2}} \right)^{2}B^{2}}+\frac{R_{0}\tanh\frac{B}{B_{AHE}}}{B}\#\left( S16 \right) \end{aligned}$$

where the first term describes the conventional multiband effect with $j=1,2,3\ldots$ labeling subband index, and the second term describes the AHE effect as in reference [8]. Below, we focus on the $R_{H}$ data shown in the main text, especially the dip-to-peak transition in the low-field region and discuss three possible scenarios. We check the goodness of fitting in these scenarios by using the Levenberg-Marquardt nonlinear curve fitting algorithm with constraint $R_{S}^{-1}\left( B=0 \right)=\sum e\left| n_{j}u_{j} \right|$.

1. Multiband model with hole-to-electron transition (2e+e/h)

The Rashba model indicates the existence of a Dirac point marking hole-to-electron transition, which can naturally explain the dip-to-peak feature in $R_{H}$. We test this scenario by including 3 bands with two electron bands (by restricting $n_{1},n_{2}>0$) and one hole band (by allowing $n_{3}$ to change sign upon gating) and drop the AHE term (by setting $R_{0}=0$). However, no reliable fitting can be found in the dip-to-peak transition range of $V_{\mathrm{bg}}$, as shown in Fig. S9(a), suggesting this scenario is not responsible for the transport data.

1. Multiband model with AHE (2e+AHE)

The dip feature in *R*_H_ has been studied in detail by Gunkel et al. in reference [8] by introducing AHE to account for the dip feature in the Bell shape background in $R_{H}$ in NGO/STO samples. Although our samples show no sign of magnetic hysteresis (Fig. S8), we still test this scenario by including 2 electron bands and AHE in Eq. (S16). As a result, no reasonable fitting parameters can be found either (Fig. S9(b)), suggesting AHE is unlikely to be present in our samples.

1. Multiband model with quantum corrections (2e+Q)

The quantum process that involves TRS protection, e.g., the WAL process, effectively avoids scattering and increases carrier mobility through destructive interference of electron wavefunctions. Since the magnetic field destroys TRS, this mobility enhancement is suppressed with increasing magnetic field. For the TRS-protected transport on FDWs described in the main text, it is thus plausible to assume that a magnetic field dependent mobility. The best fitting that faithfully matches the data is when an exponential dependence of mobility and magnetic field is assumed. Here we include 2 electron bands ($n_{1},\mu_{1}$) and ($n_{2},\mu_{2}$), and a quantum corrected band ($n_{3},\mu_{3}$) with mobility $\mu_{3}(B)=\mu_{3}^{0}\exp(-\frac{B}{B_{c}})$. As shown in Fig. S9(c), the fitting shows excellent agreement with the data, suggesting the validity of TRS-protected transport.

To justify the fit goodness of different models, we use the root mean square error RMSE=$\sqrt{\frac{1}{n}\sum_{i=1}^{n} {{(y}_{i}-\hat{y}_{i})}^{2}}$, where $y_{i}$ is the $R_{H}$ data and $\hat{y}_{i}$ is the predicted $R_{H}$ value, to qualify the fit error. The RMSE of the different model fittings are shown in Fig. S10. The fitting process of the multiband model with quantum corrections (2e+Q) yields the smallest RMSE, demonstrating that it provides the best fit to the data.

We note the quenching of the TRS-protected subband causes the overall Hall mobility to quickly drop from over 20,000 cm^2^/Vs at low fields to ~2,000 cm^2^/Vs at high magnetic fields. This reduced mobility can potentially explain that the QOs are not observed at the high magnetic field.

We also note the reference [9] describes that a universal Lorentzian scaling law exists at the LAO/STO interface related to the Lifshitz transition from Ti $d_{xy}$ orbital to $d_{xz}$, $d_{yz}$ orbitals. We performed a similar analysis of the Hall coefficient data by fitting to $R_{H}=R_{\infty}+\frac{R_{0}-R_{\infty}}{1+\left( B/{B_{W}} \right)^{2}}$ , where $R_{0}$ and $R_{\infty}$ is the value of $R_{H}$ at zero and infinite magnetic fields, respectively, and $B_{W}$is the characteristic

field. The fitting results (Fig. S12) show scaled $R_{H}$ of $V_{\mathrm{bg}}$ do not fall under a universal scaling law, which suggests that the orbital characteristics of our samples are different from regular low-mobility LAO/STO samples.

CORRELATED PHASE DIAGRAM

Sample A was cooled to ~100 mK to study the relationship between superconductivity and anomalous quantum oscillations. Sample A is superconducting under relatively low $V_{\mathrm{bg}}$ conditions. Fig. S13(a) shows the data of temperature-dependent 4 terminal resistance (in configuration A) and quantum oscillations at *T*=100 mK as a function of back gate voltages. The critical temperature $T_{c}$ is defined by the 10% drop of the normal resistance at 230 mK. According to the evolution of $T_{c}$ as a function of $V_{\mathrm{bg}}$, superconductivity almost disappears over 20 V while quantum oscillations gradually begin to emerge, indicating a transition between superconductivity and quantum oscillations takes place over 20 V~50 V. The phase diagram of sample A as a function of temperature and $V_{\mathrm{bg}}$ is shown in Fig. S14.


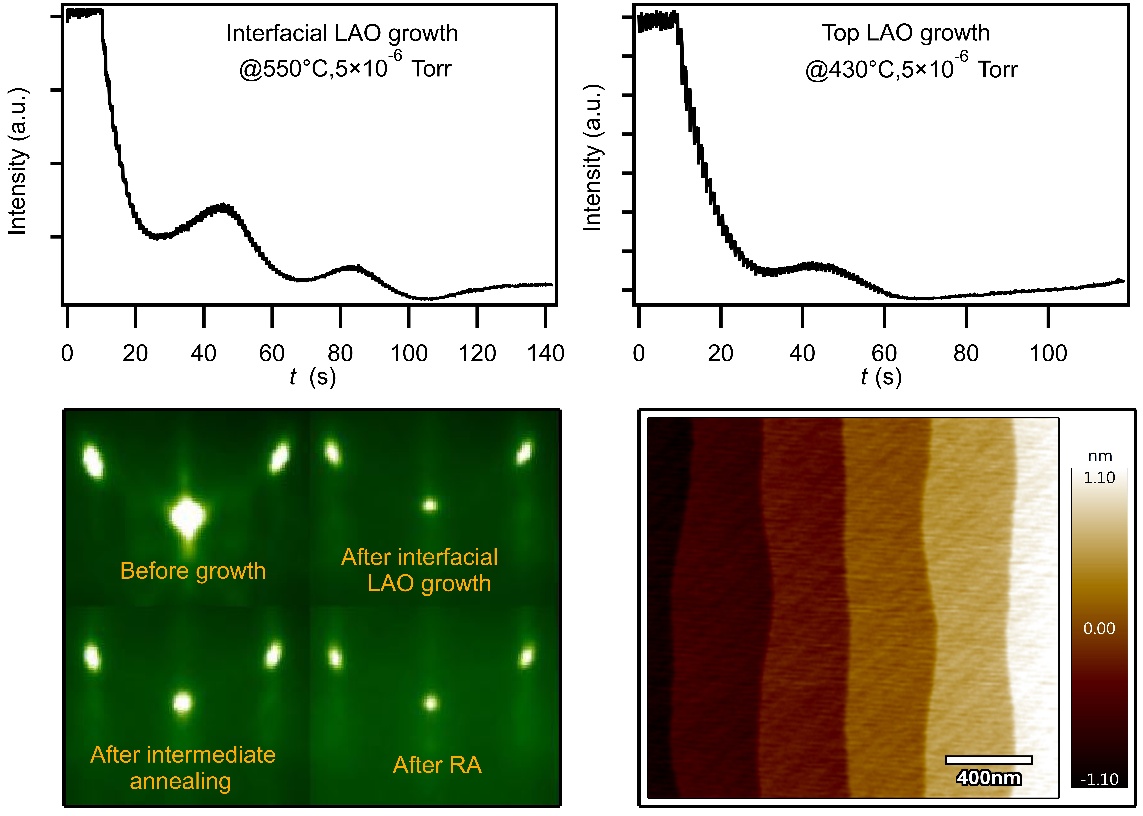


**Fig. S1.** **(*m*+*n*) LAO/STO sample growth.** (A) and (B) show RHEED oscillations for the interfacial *m* uc and top *n* uc LAO growth. (C) RHEED patterns at various growth stages. (D) AFM image shows an atomically flat surface after growth.


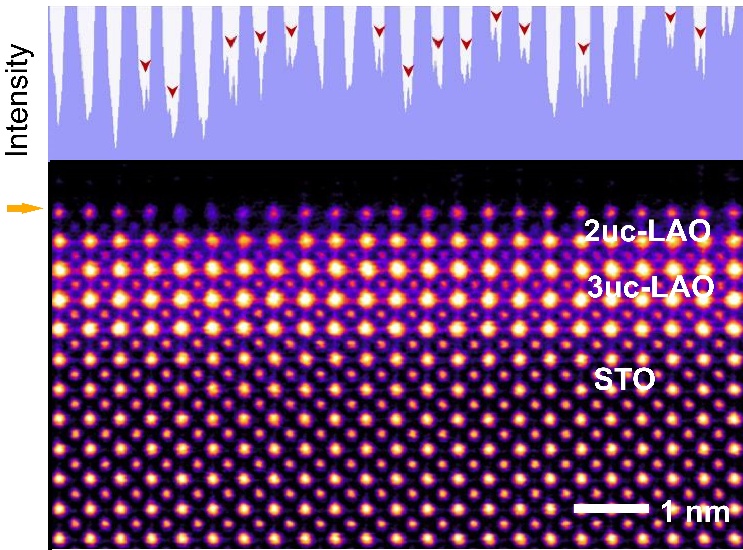


**Fig. S2.** **ADF-STEM imaging of sample A.** Red arrows in the top panel mark oxygen peaks (with some columns missing due to oxygen deficiency) along the atomic plane indicated by the yellow arrow.


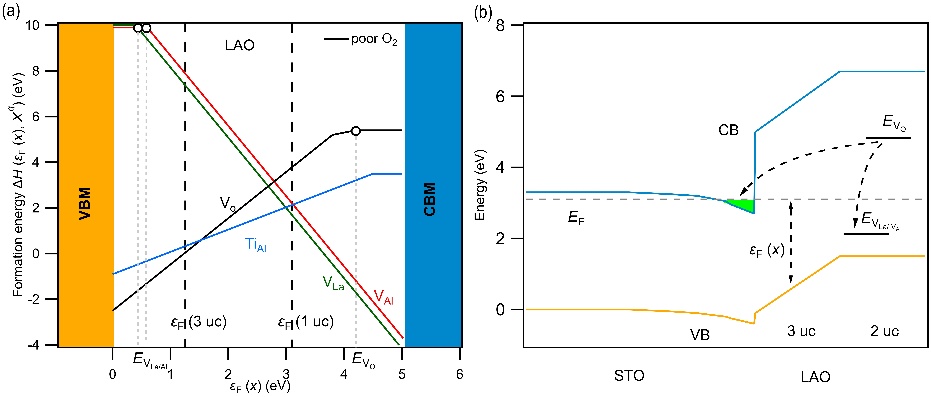


**Fig. S3. Energy diagrams calculated by DFT.** (a) The formation energy diagram of the intrinsic defects and the $\mathrm{Ti}_{\mathrm{Al}}$ defect in LAO, where the solid polylines are calculated with the O_2_-poor ($\Delta\mu_{O}=-2 eV$) growth condition. The vertical dashed line represents the Fermi levels relative to the VBM at the 1 uc and 3 uc of the LAO slab. The open circles indicate the defect charge transition energy levels $E_{V_{La/Al}}$and $E_{V_{O}}$. (b) The band alignment schematic of 5 uc LAO slab on STO substrate, where the horizontal dashed line represents the Fermi level of the sample.


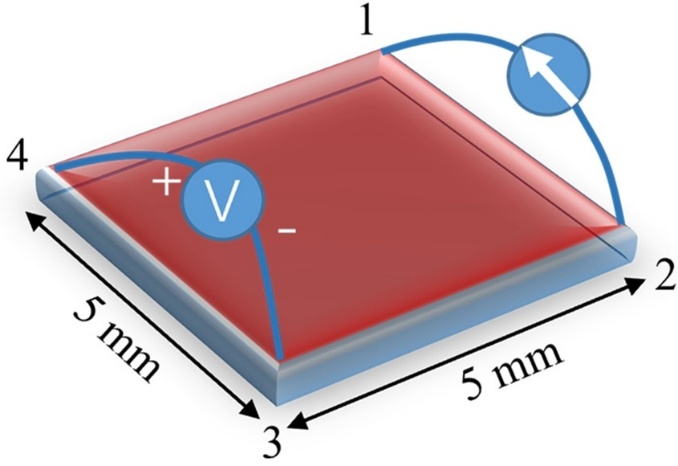


**Fig. S4. Configuration A of the spinning current Hall measurement.** Contact 1 and 2 are connected to source current. Contact 3 and 4 are connected to a voltmeter.


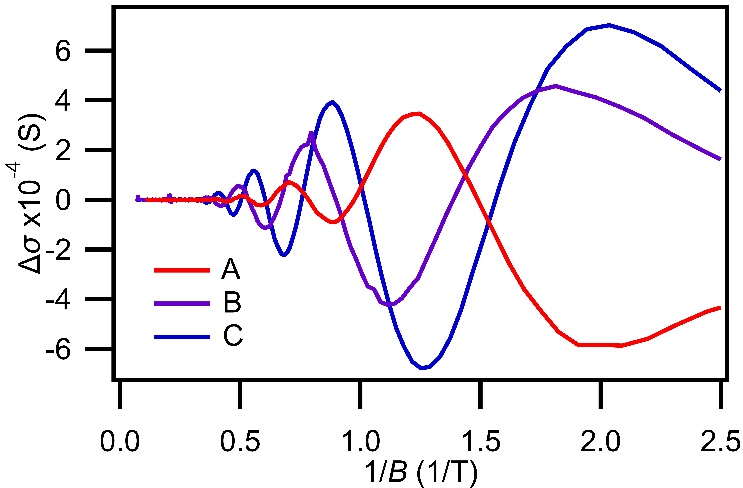


Fig. S5. Conductance plot of quantum oscillations in Sample A, B and C. The oscillation is aperiodic in 1/B axis, and the oscillation amplitude decreases with increasing magnetic field.


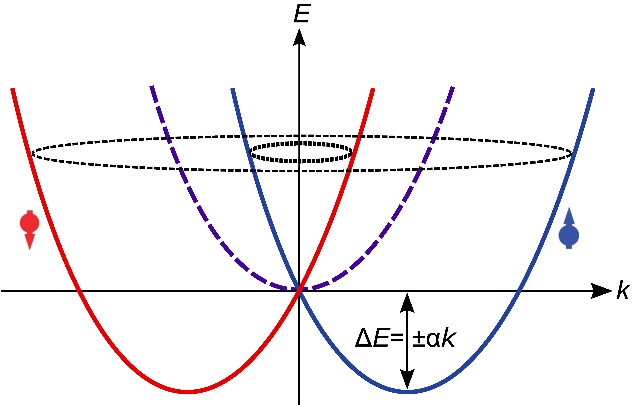


**Fig. S6. Rashba model on FDWs.** Two Fermi pockets emerge with the inclusion of the linear Rashba SOC in the Hamiltonian.


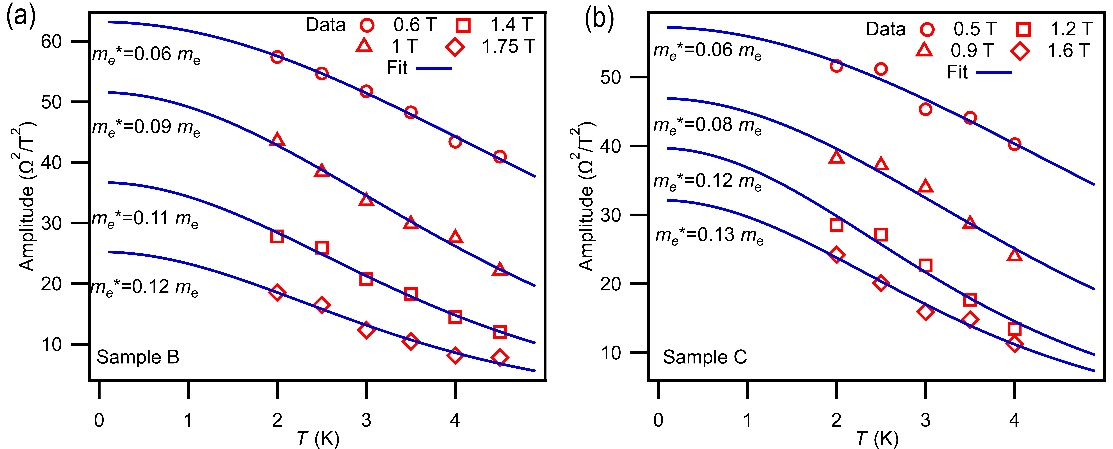


**Fig. S7. Effective mass extraction for Sample B and C.** Extended L-K model fitting of temperature-dependent oscillations in ${d^{2}R_{s}}/{dB^{2}}$ for Sample B (a) and Sample C (b).


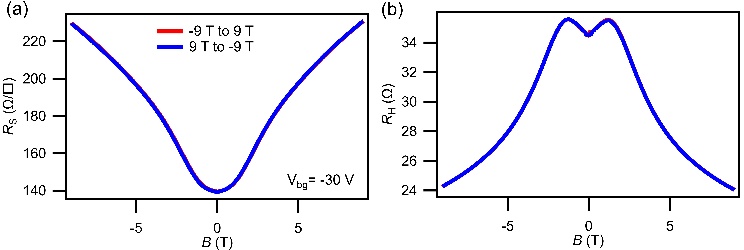


Fig. S8. No hysteresis is observed during consecutive magnetic field sweeps from -9 T to 9 T, then back to -9 T in both $\boldsymbol{R}_{\mathbf{S}}$ (a) and $\boldsymbol{R}_{\mathbf{H}}$ (b).


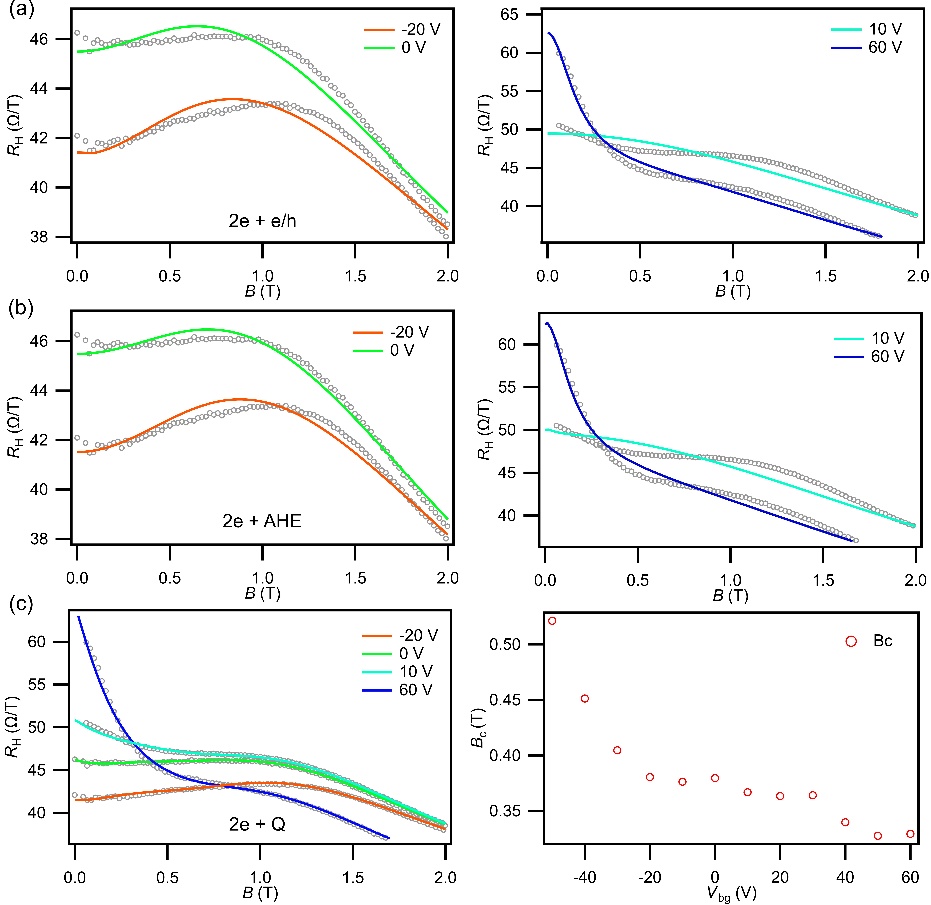


**Fig. S9.** $\boldsymbol{R}_{\boldsymbol{H}}$ **fitting by the multiband model with (a) hole-to-electron transition (2e+e/h), (b)AHE (2e+AHE) and (c) quantum corrections (2e+Q).** $R_{H}$ data (grey circles) and fitting results of 2e+e/h model and 2e+AHE model for -20 V (orange line), 0 V (green line), 10 V (cyan line), and 60 V (blue line) $V_{\mathrm{bg}}$ show clear deviation in the low magnetic field region. While fitting results of 2e+Q model shows excellent agreement. The best-fitting parameters are shown in Fig. 4 in the main text. The right (c) shows the extracted characteristic magnetic field in the field dependence of $\mu_{3}(B)=\mu_{3}^{0}\exp(-\frac{B}{B_{c}})$.


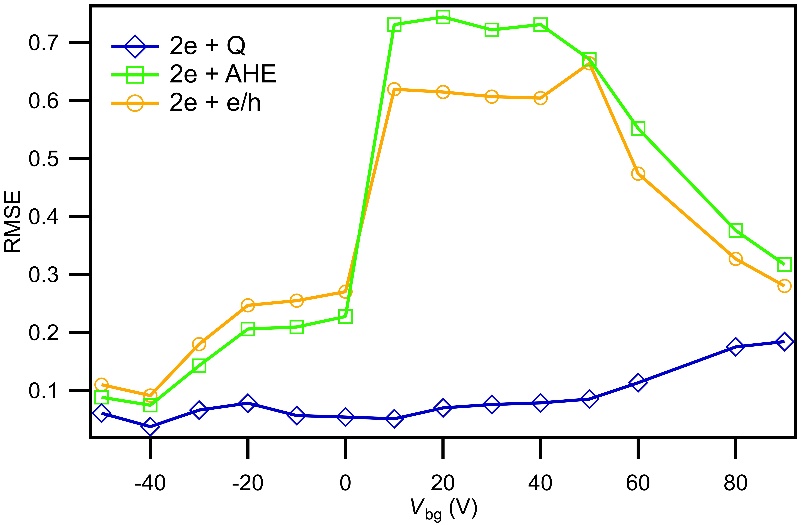


Fig. S10. Goodness of fit. The RMSEs of fitting the gate-tunable R_H_ data are shown for three models: 2e+h/e (red circle), 2e+AHE (green square), and 2e+Q (blue diamond). The quantum-corrected model (2e+Q) shows the best fitting.


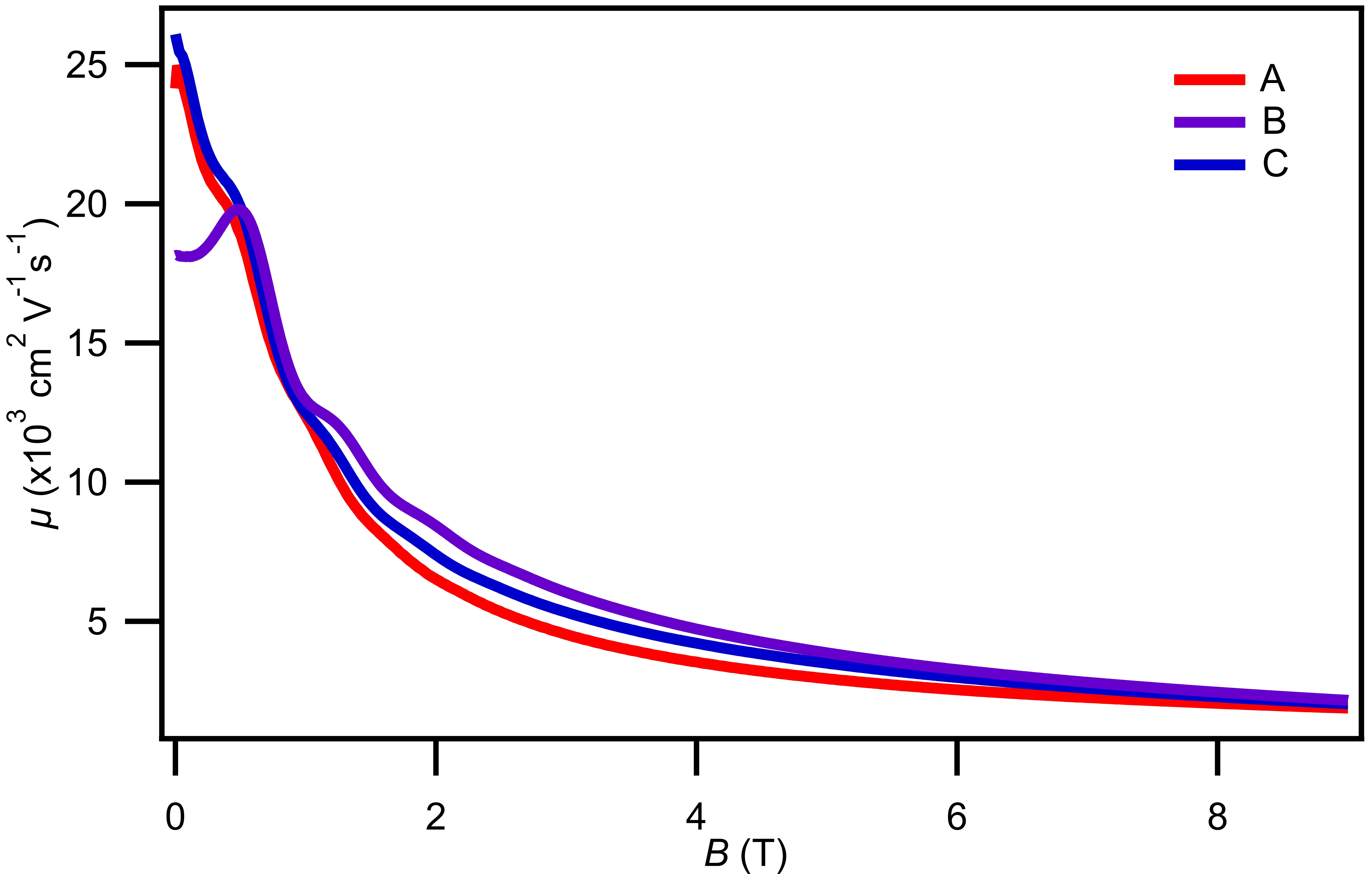


Fig. S11. Magnetic field dependence of low-field Hall mobility (weighted average of all subbands) of sample A-C. The mobility quickly drops from over 20,000 cm^2^/Vs to only a few thousand with increasing magnetic field.


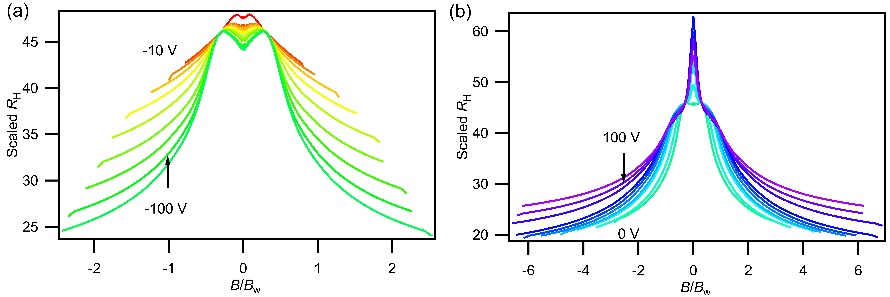


Fig. S12. Failure of the universal Lorentzian scaling. (a) and (b) show scaled $\boldsymbol{R}_{\mathbf{H}}$ of curves over $\boldsymbol{-100}\mathbf{V}\boldsymbol{<}\boldsymbol{V}_{\mathbf{bg}}\boldsymbol{<-10}\mathbf{V}$ and $\boldsymbol{0}\mathbf{V}\boldsymbol{<}\boldsymbol{V}_{\mathbf{bg}}\boldsymbol{<100}\mathbf{V}$ ranges, respectively.


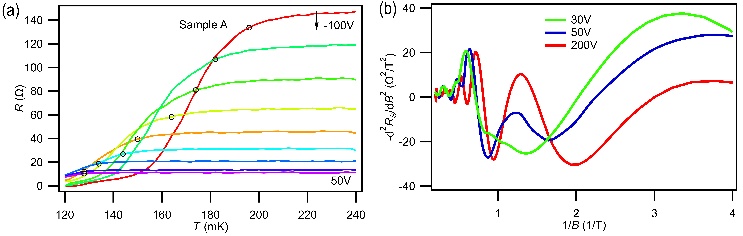


**Fig. S13. Gate-tunable (a) superconductivity and (b) quantum oscillations of samples A.** Gate voltages vary from -100 V to 50 V.

**Fig. S14. Correlated phase diagram of sample A as a function of temperature and** $\boldsymbol{V}_{\mathbf{bg}}$**.** The transition from superconductivity to anomalous quantum oscillations takes place at a $V_{\mathrm{bg}}\sim50 V$, suggesting possible existence of a quantum critical point. The error bar ($\sim20 V$) in $V_{\mathrm{bg}}$ marks the transition range of oscillations from emerging to fully developed.


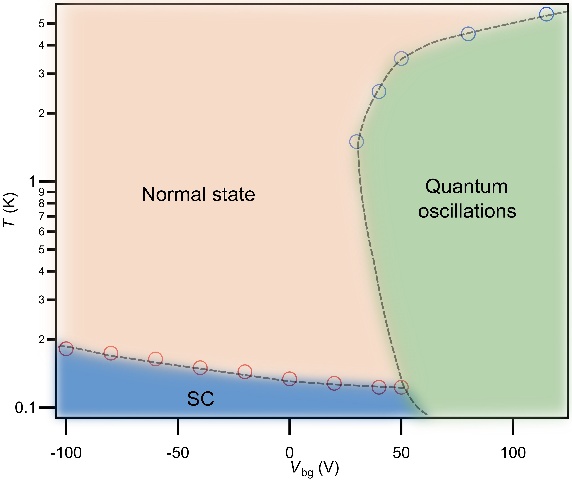


**Table S1. Configuration setup of the spinning current Hall measurement.** For every configuration, current enters the sample at contact “*I*_+_” and leaves at contact “*I*_-_”, voltage difference is measured between contact “*V*_+_” and “*V*_-_”. The measured voltage consists of 3 contributions: Hall voltage, longitudinal voltage and thermoelectric voltage. For vdP configurations (A, B, C and D), the small Hall voltage part ($\pm c_{1,2}R_{H}BI$, $\left| c_{1,2} \right|\ll1$) is due to misalignment. For Hall configurations (E, F, G and H), misalignment leads to a small longitudinal voltage contribution ($\pm\alpha R_{s}I$, $\left| \alpha\right|\ll1$). Thermoelectric voltage $V_{\mathrm{th}}$ is eliminated by calculating the slope of *I*-*V* curve, which is defined as the measured resistance of a single *I*-*V* curve measurement.

| Configuration | Contact name | | | | Measured voltage $V$ | | | $dV/dI$ |
| --- | --- | --- | --- | --- | --- | --- | --- | --- |
|  | *I*_+_ | *I*_-_ | *V*_+_ | *V*_-_ | Hall voltage | Longitudinal voltage | Thermoelectric voltage |  |
| A | 1 | 2 | 4 | 3 | $c_{1}R_{H}BI$ | $R_{a}I$ | $V_{\mathrm{th}}^{43}$ | $R_{12,43}=R_{a}+c_{1}R_{H}B$ |
| B | 2 | 3 | 1 | 4 | $c_{2}R_{H}BI$ | $R_{b}I$ | $V_{\mathrm{th}}^{14}$ | $R_{23,14}=R_{b}+c_{2}R_{H}B$ |
| C | 3 | 4 | 2 | 1 | $-c_{1}R_{H}BI$ | $R_{a}I$ | $V_{\mathrm{th}}^{21}$ | $R_{34,21}=R_{a}-c_{1}R_{H}B$ |
| D | 4 | 1 | 3 | 2 | $-c_{2}R_{H}BI$ | $R_{b}I$ | $V_{\mathrm{th}}^{32}$ | $R_{41,32}=R_{b}-c_{2}R_{H}B$ |
| E | 1 | 3 | 4 | 2 | $R_{H}BI$ | $\alpha R_{s}I$ | $V_{\mathrm{th}}^{42}$ | $R_{13,42}=R_{H}B+\alpha R_{s}$ |
| F | 2 | 4 | 1 | 3 | $R_{H}BI$ | $-\alpha R_{s}I$ | $V_{\mathrm{th}}^{13}$ | $R_{24,13}=R_{H}B-\alpha R_{s}$ |
| G | 3 | 1 | 2 | 4 | $R_{H}BI$ | $\alpha R_{s}I$ | $-V_{\mathrm{th}}^{42}$ | $R_{13,42}=R_{H}B+\alpha R_{s}$ |
| H | 4 | 2 | 3 | 1 | $R_{H}BI$ | $-\alpha R_{s}I$ | $-V_{\mathrm{th}}^{13}$ | $R_{13,42}=R_{H}B-\alpha R_{s}$ |

**Table S2.** Subband parameters extracted from the analysis of the generalized Lifshitz–Onsager quantization rule and Rashba model.

| Sample | $m_{e}^{*}$($m_{e}$) | $Ω_{F}$ ($s^{-1}$) | $l_{y}$ ($nm$) | | $\alpha_{R} (eVm)$ | $d_{c} (nm)$ | $W (nm)$ |
| --- | --- | --- | --- | --- | --- | --- | --- |
| A | 0.04@0.5 T  0.07@1.1 T  0.08@1.4 T | $2.4\times{10}^{13}$ | 21 | $1.1\times{10}^{-10}$@1.4 T | | 218@0.5 T  104@2.2 T | 83 |
| B | 0.06@0.6 T  0.09@1.0 T | $2.4\times{10}^{13}$ | 17 | $1.3\times{10}^{-10}$@1.0 T | | 220@0.6 T  100@2.9 T | 70 |
| C | 0.06@0.5 T  0.08@0.9 T | $2.8\times{10}^{13}$ | 17 | $1.4\times{10}^{-10}$@0.9 T | | 262@0.5 T  115@2.6 T | 74 |
|  |  |  |  |  | |  |  |

**References**

1. Liu X, Zhou T, Qin Z*, et al*. Nonlinear optical phonon spectroscopy revealing polaronic signatures of the LaAlO_3_/SrTiO_3_ interface. *Sci. Adv.* 2023; **9**: eadg7037.

2. Yu L, Zunger A. A polarity-induced defect mechanism for conductivity and magnetism at polar–nonpolar oxide interfaces. *Nat. Commun.* 2014; **5**: 5118.

3. Sample H, Bruno W, Sample S*, et al*. Reverse‐field reciprocity for conducting specimens in magnetic fields. *J. Appl. Phys.* 1987; **61**: 1079-1084.

4. Mosser V, Matringe N, Haddab Y. A spinning current circuit for Hall measurements down to the nanotesla range. *IEEE Trans. Instrum. Meas.* 2017; **66**: 637-650.

5. Shoenberg D. *Magnetic Oscillations in Metals*. Cambridge University Press, 1984.

6. Berggren KF, Roos G, van Houten H. Characterization of very narrow quasi-one-dimensional quantum channels. *Phys. Rev. B* 1988; **37**: 10118-10124.

7. Veit MJ, Arras R, Ramshaw BJ*, et al*. Nonzero Berry phase in quantum oscillations from giant Rashba-type spin splitting in LaTiO_3_/SrTiO_3_ heterostructures. *Nat. Commun.* 2018; **9**: 1458.

8. Gunkel F, Bell C, Inoue H*, et al*. Defect Control of Conventional and Anomalous Electron Transport at Complex Oxide Interfaces. *Phys. Rev. X* 2016; **6**: 031035.

9. Joshua A, Pecker S, Ruhman J*, et al*. A universal critical density underlying the physics of electrons at the LaAlO_3_/SrTiO_3_ interface. *Nat. Commun.* 2012; **3**: 1129.
