# Supplementary material for: Time reversal symmetry-protected transport at correlated oxide interfaces
Source: Natl Sci Rev. 2025 May 7;12(6):nwaf156. doi: 10.1093/nsr/nwaf156 (PMC12163994; doi:10.1093/nsr/nwaf156)
Supplement: nwaf156_Supplemental_Files [file nwaf156_supplemental_files.zip › Tables_Supplementary data.docx]

**Table S1. Configuration setup of the spinning current Hall measurement.** For every configuration, current enters the sample at contact “*I*_+_” and leaves at contact “*I*_-_”, voltage difference is measured between contact “*V*_+_” and “*V*_-_”. The measured voltage consists of 3 contributions: Hall voltage, longitudinal voltage and thermoelectric voltage. For vdP configurations (A, B, C and D), the small Hall voltage part ($\pm c_{1,2}R_{H}BI$, $\left| c_{1,2} \right|\ll1$) is due to misalignment. For Hall configurations (E, F, G and H), misalignment leads to a small longitudinal voltage contribution ($\pm\alpha R_{s}I$, $\left| \alpha\right|\ll1$). Thermoelectric voltage $V_{\mathrm{th}}$ is eliminated by calculating the slope of *I*-*V* curve, which is defined as the measured resistance of a single *I*-*V* curve measurement.

| Configuration | Contact name | | | | Measured voltage $V$ | | | $dV/dI$ |
| --- | --- | --- | --- | --- | --- | --- | --- | --- |
|  | *I*_+_ | *I*_-_ | *V*_+_ | *V*_-_ | Hall voltage | Longitudinal voltage | Thermoelectric voltage |  |
| A | 1 | 2 | 4 | 3 | $c_{1}R_{H}BI$ | $R_{a}I$ | $V_{\mathrm{th}}^{43}$ | $R_{12,43}=R_{a}+c_{1}R_{H}B$ |
| B | 2 | 3 | 1 | 4 | $c_{2}R_{H}BI$ | $R_{b}I$ | $V_{\mathrm{th}}^{14}$ | $R_{23,14}=R_{b}+c_{2}R_{H}B$ |
| C | 3 | 4 | 2 | 1 | $-c_{1}R_{H}BI$ | $R_{a}I$ | $V_{\mathrm{th}}^{21}$ | $R_{34,21}=R_{a}-c_{1}R_{H}B$ |
| D | 4 | 1 | 3 | 2 | $-c_{2}R_{H}BI$ | $R_{b}I$ | $V_{\mathrm{th}}^{32}$ | $R_{41,32}=R_{b}-c_{2}R_{H}B$ |
| E | 1 | 3 | 4 | 2 | $R_{H}BI$ | $\alpha R_{s}I$ | $V_{\mathrm{th}}^{42}$ | $R_{13,42}=R_{H}B+\alpha R_{s}$ |
| F | 2 | 4 | 1 | 3 | $R_{H}BI$ | $-\alpha R_{s}I$ | $V_{\mathrm{th}}^{13}$ | $R_{24,13}=R_{H}B-\alpha R_{s}$ |
| G | 3 | 1 | 2 | 4 | $R_{H}BI$ | $\alpha R_{s}I$ | $-V_{\mathrm{th}}^{42}$ | $R_{13,42}=R_{H}B+\alpha R_{s}$ |
| H | 4 | 2 | 3 | 1 | $R_{H}BI$ | $-\alpha R_{s}I$ | $-V_{\mathrm{th}}^{13}$ | $R_{13,42}=R_{H}B-\alpha R_{s}$ |

**Table S2.** Subband parameters extracted from the analysis of the generalized Lifshitz–Onsager quantization rule and Rashba model.

| Sample | $m_{e}^{*}$($m_{e}$) | $Ω_{F}$ ($s^{-1}$) | $l_{y}$ ($nm$) | | $\alpha_{R} (eVm)$ | $d_{c} (nm)$ | | $W (nm)$ |  |
| --- | --- | --- | --- | --- | --- | --- | --- | --- | --- |
| A | 0.04@0.5 T  0.07@1.1 T  0.08@1.4 T | $2.4\times{10}^{13}$ | 21 | $1.1\times{10}^{-10}$@1.4 T | | | 218@0.5 T  104@2.2 T | 83 | |
| B | 0.06@0.6 T  0.09@1.0 T | $2.4\times{10}^{13}$ | 17 | $1.3\times{10}^{-10}$@1.0 T | | | 220@0.6 T  100@2.9 T | 70 | |
| C | 0.06@0.5 T  0.08@0.9 T | $2.8\times{10}^{13}$ | 17 | $1.4\times{10}^{-10}$@0.9 T | | | 262@0.5 T  115@2.6 T | 74 | |
|  |  |  |  |  | | |  |  | |
